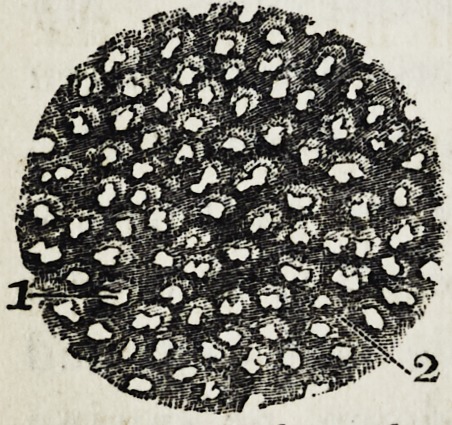# Micrology.—No. 1

**Published:** 1879-05

**Authors:** George B. Harriman

**Affiliations:** Boston, Mass.


### ARTICLE III.

### *Department of Micrology.*—No. 1.

BY GEORGE B. HARRIMAN, D. D. S., M. D., BOSTON, MASS.

The industrious student in Micrology is always making advances in the sciences that he carefully investigates.

The old theory of the tubulous structure of dentine was advanced by Leuwenhock about two hundred years ago. Later investigations by Retsius, Harris and McQuillen and others, advanced the same theories or notions, unsubstantiated by facts, and it is a question in my mind whether or not they really investigated the subject carefully, or merely copied what Leuwenhock had written two hundred years before.

In 1868 I commenced the microscopical examinations of the teeth. The reader will find the results of some of my investigations in the May No. of the American Journal of Dental Science, 1870. In that number I stated my process of preparing (page 3) in these words: "It may be interesting to the reader to be made acquainted with the process. These sections can be made of any desired size, and attenuated to any point from the one-two hundredth to the one-three thousandth of an inch in thickness. My process is to glue the tooth into a piece of wood, and fasten the wood in a lathe where there is a carriage which runs in a rack or pinion, with a tool post to guide the cutting instrument. In this way I obtain a section after my own desire." The process is exceedingly simple and easy, and consumes much less time than grinding down or sawing sections thin enough to be thoroughly and successfully examined with the microscope. By turning off a thin shaving with the lathe, I was able at least eleven years ago to show that the dentine was composed of cells and fibres of soft-solid substance, instead of being "tubes filled with a fluid."

The following cut shows how a thin shaving turned off by the above process looks when placed on a glass slide and magnified about five hundred diameters, and treated with acetic acid.

Figure 1 in the cut shows a mass of soft-solid substance, or an aggregation of cells with nuclei. No. 2 shows the cells closely joined together. No. 3 shows the lime salts, or interstitial substance.

I think Dr. Puffer, of Bridgewater, Mass., was present and witnessed the action of the acid upon the section, with me. We saw the lime salts dissolve and the cells and fibres swell up like a dried cork immersed in hot water.

Cut No. 2 represents a thin section of tooth treated with a strong solution of caustic potassa, disorganizing nearly all of the animal substance.

Figure 1 points to the vacant cavity where the soft, solid substance has been destroyed by the action of the caustic solution. No. 2 points to the lime salts which is somewhat blackened by the action of the chemical, and is magnified about one thousand diameters. Much care should be taken in treating thin sections of dentine in this way. The investigator will find it much more difficult to treat successfully a section of dentine in this way, than by the former process. In this operation the dentine is liable to break and crumble, much to the annoyance of the experimenter, but perseverance will accomplish wonders.

The chemist tells us that the substance of the tooth is composed of one-third animal matter and two-thirds mineral substance. Where does the animal matter come from, if the so called "tubes" are not filled with animal matter?

## Figures and Tables

**Figure f1:**
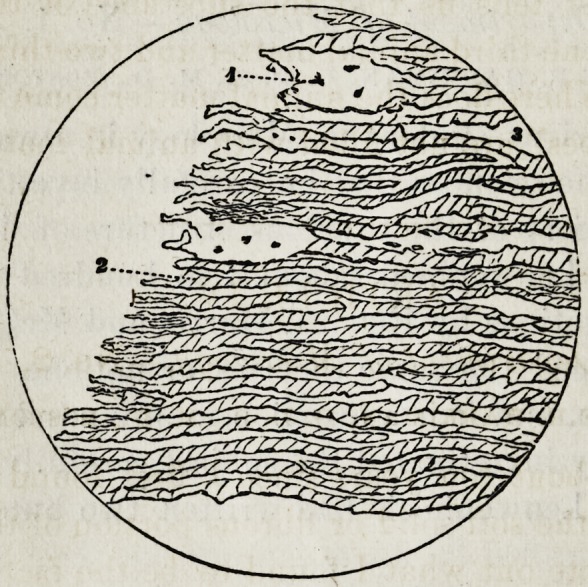


**Figure f2:**